# Melanin Biopolymers in Pharmacology and Medicine—Skin Pigmentation Disorders, Implications for Drug Action, Adverse Effects and Therapy

**DOI:** 10.3390/ph17040521

**Published:** 2024-04-18

**Authors:** Marta Karkoszka, Jakub Rok, Dorota Wrześniok

**Affiliations:** Department of Pharmaceutical Chemistry, Faculty of Pharmaceutical Sciences in Sosnowiec, Medical University of Silesia, Jagiellońska 4, 41-200 Sosnowiec, Poland; dwrzesniok@sum.edu.pl

**Keywords:** melanin, melanocytes, drug–melanin complex, hyperpigmentation, hypopigmentation

## Abstract

Melanins are biopolymeric pigments formed by a multi-step oxidation process of tyrosine in highly specialized cells called melanocytes. Melanin pigments are mainly found in the skin, iris, hair follicles, and inner ear. The photoprotective properties of melanin biopolymers have been linked to their perinuclear localization to protect DNA, but their ability to scavenge metal ions and antioxidant properties has also been noted. Interactions between drugs and melanins are of clinical relevance. The formation of drug–melanin complexes can affect both the efficacy of pharmacotherapy and the occurrence of adverse effects such as phototoxic reactions and discoloration. Because the amount and type of melanin synthesized in the body is subject to multifactorial regulation—determined by both internal factors such as genetic predisposition, inflammation, and hormonal balance and external factors such as contact with allergens or exposure to UV radiation—different effects on the melanogenesis process can be observed. These factors can directly influence skin pigmentation disorders, resulting in hypopigmentation or hyperpigmentation of a genetic or acquired nature. In this review, we will present information on melanocyte biology, melanogenesis, and the multifactorial influence of melanin on pharmacological parameters during pharmacotherapy. In addition, the types of skin color disorders, with special emphasis on the process of their development, symptoms, and methods of treatment, are presented in this article.

## 1. Introduction

Skin color is the result of the presence of three pigments: brown-black or yellow-red biopolymers called melanins; hemoglobin present in red blood cells; and carotenoids [1]. Melanins are biopolymers produced by highly specialized pigment cells called melanocytes. Tge high specialization of melanin-producing cells and the complex structure of melanin biopolymers contribute to the diversity of functions of these natural pigments. In addition to their pigmentation function, melanins protect cells against the harmful effects of solar radiation, free radicals, and even some toxic substances. Part of the cytoprotective properties stem from the ability of melanins to bind chemicals by forming stable complexes. It should be noted, however, that the process of complex formation can also affect the action of both locally and systemically administered drugs. On one hand, drugs bound to melanin lack pharmacodynamic properties and cannot directly interact with receptors or enzymes. On the other hand, the formation of such complexes may lead to the preferential accumulation of drugs in pigmented tissues, thereby increasing the risk of side effects.

The process of melanogenesis itself is also particularly interesting from a medical standpoint. Disorders in melanin synthesis form the basis for hypo- or hyperpigmentation diseases. The complexity of pigmentation disorders largely results from the multidirectional regulation of the melanogenesis process. It should be emphasized that the course of melanin synthesis is influenced by both internal factors, including genetic and hormonal factors, and external factors, such as UV radiation or exogenous substances. This article provides a summary of the key information regarding the biological functions of melanin and melanocytes, with consideration of pharmacological and medical aspects.

## 2. The Basis of Melanocyte Biology

Pigment cells were first described in 1819 by Giosue Sangiovanni, who discovered their presence in squid and named them chromatophores. Eighteen years later, German anatomist and pathologist Jakob Henle identified pigment cells in the human epidermis, which turned out to be identical to the pigment cells found in the eye. The term “melanin” comes from the Greek word “melas”, meaning “black” or “dark”, and was introduced by the French anatomist and biologist Charles-Philippe Robin in 1873. In turn, the term “melanocyte” was introduced in 1889 by Samuel Meyerson [2].

**Figure 1:**
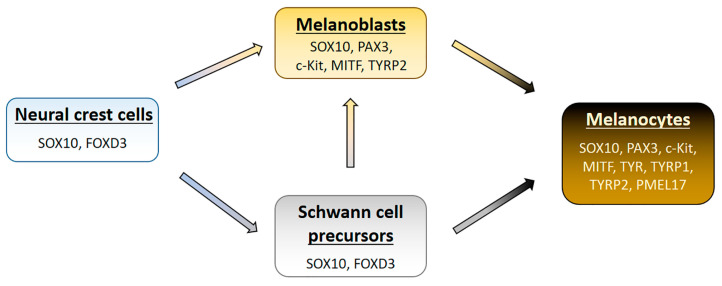
Diagram showing the development of melanocytes. Characteristic molecular markers are placed under the cell names.

Melanocytes are of neuroectodermal origin and are derived from multipotent neural crest cells (NCCs) (Figure 1) [3,4,5,6]. The early induction of NCC development primarily occurs under the influence of BMP (bone morphogenetic protein) group cytokines. Immediately after induction, NCCs undergo epithelial–mesenchymal transition (EMT), during which they lose adhesion to surrounding cells and migrate. The EMT process is mainly regulated by transcription factors Snail/Slug, which, by inhibiting E-cadherin expression, enable the detachment of NCCs [7,8]. It should be mentioned that melanocytes in the skin may originate not only directly from migrating neural crest cells but also by detaching from the nerves innervating the skin as well as arising from the differentiation of so-called Schwann cell precursors (SCPs) located in the skin nerves [9,10,11]. SCPs reach the epidermis during fetal development during the process of skin innervation and can differentiate into Schwann cells, intraneural fibroblasts, or melanocytes.

Precursor cells for melanocytes, called melanoblasts, are formed with the involvement of the Wnt signaling pathway [6]. Although melanoblasts are non-pigmented cells, they possess molecular markers characteristic of melanocytes, such as the c-Kit receptor with tyrosine kinase activity, the microphthalmia-associated transcription factor (MITF), transcription factors SOX10 and PAX3, and tyrosinase-related protein 2 (TYRP2) [3,9,12]. The formation of melanoblasts from SCPs is influenced, among other factors, by an increase in levels of IGF (insulin-like growth factor) and PDGF (platelet-derived growth factor) produced by both immature and mature Schwann cells [6]. Due to the potential for Schwann cell differentiation in vitro, they can transform into melanocytes even after birth [3,13].

The migration of melanoblasts to their target locations occurs between the 6th and 8th weeks of embryonic development, and by the 12th to 13th week, most of the cells are already in the epidermis. During migration, melanoblasts proliferate and begin to express genes related to melanogenesis, primarily under the influence of the transcription factor MITF. Upon reaching their target locations, melanoblasts differentiate into melanocytes. One of the key factors determining the maturation of melanocytes is the activity of tyrosinase, a key enzyme in the melanin biosynthesis process [3].

Mature melanocytes are dendritic cells with numerous extensions, although they can also assume a more oval shape. The formation of dendritic extensions is primarily influenced by cytoskeletal elements: microtubules provide structural support, and actin filaments are responsible for their growth. In humans, melanocytes are mainly found in the epidermis, hair follicles, iris, and choroid of the eye, where they impart color to these structures through melanin production. Additionally, melanocytes have been found in the inner ear, central nervous system, heart, and lungs. It is worth noting that besides melanocytes, cells capable of melanin synthesis include retinal pigment epithelium (RPE) cells, iris epithelium, and ciliary body of the eye, as well as some neurons and adipocytes [3,14].

## 3. Melanosomes—Biogenesis, Structure, and Function

Melanosomes belong to organelles associated with lysosomes, known as LROs (lysosome-related organelles). LROs are a group of tissue-specific cellular structures surrounded by a membrane that shares common characteristics with regular lysosomes, such as their origin from the endocytic system and the presence of lysosomal proteins [15]. The biogenesis and maturation of melanosomes consist of four stages [16,17,18]:

- Stage 1—called premelanosomes, with a spherical shape, lacking both internal structural proteins and tyrosinase; at this stage, the organization of the melanosomal matrix begins.
- Stage 2—elongated, ellipsoidal melanosomes with tyrosinase and an internal fibrillar matrix, primarily composed of PMEL17 protein.
- Stage 3—melanosomes are partially filled with melanin, which is deposited on the fibrils of the melanosomal matrix.
- Stage 4—melanosomes are characterized by low tyrosinase activity and a high melanin content, which obscures the internal structure.

It should be clearly noted that cells synthesizing pheomelanin are characterized by a strong downregulation of PMEL17 expression by agouti signaling. As a result, pheomelanosomes lack a fibrillar matrix, and the organelles themselves have an irregular, spherical shape, unlike eumelanosomes [19].

During their formation, enzymes and structural proteins are delivered and incorporated into their structure in a specific order, influencing both the functional and morphological diversity of melanosomes at different developmental stages. Among the group of proteins specific to melanosomes, we can distinguish enzymes involved in the melanogenesis process (tyrosinase and tyrosinase-related proteins 1 and 2), structural proteins (e.g., premelanosome protein PMEL17, the main component of the eumelanosome matrix), and membrane proteins (e.g., P-type transporters, MATP/SLC45A2, SLC24A5, and SL7A11, which are involved in regulating pH, osmolarity as well as calcium ion and cysteine content in melanosomes, respectively) [16,19,20].

Mature melanosomes, in stage 4, are transported from the central part of skin melanocytes to the tips of dendritic extensions, first through microtubules and then via actin filaments. In the final step, melanosomes pass into keratinocytes, forming what is known as the epidermal melanin unit (EMU), where approximately 36 keratinocytes are associated with one melanocyte [21,22]. The precise mechanism of intercellular transfer of melanosomes has not been fully understood and characterized. It is postulated that it may occur through processes such as exocytosis, cytophagocytosis, fusion of the cell membranes of melanocytes and keratinocytes, and the shedding of melanosome-laden globules [17,22,23]. Most likely, various melanosome transfer mechanisms coexist and may be observed depending on physiological and pathological conditions [17]. Inside keratinocytes, melanosomes move along microtubules to the perinuclear region, where they form a kind of “microumbrella”, protecting genetic material from the harmful effects of UV radiation [24].

It is worth noting that melanosomes from light and dark skin differ in size, distribution within keratinocytes, and degree of degradation. It has been shown that melanosomes in light skin are smaller and form clusters of four to eight melanosomes associated with the membrane, while melanosomes in dark skin are mainly distributed individually. Furthermore, during keratinocyte differentiation in the upper layers of light skin, melanosomes undergo complete degradation, resulting in the absence of melanosomes in corneocytes. In the case of dark skin, melanosomes have been found to be present even in shedding corneocytes [17,25].

## 4. Structure and Biosynthesis of Melanin Biopolymers

Melanocytes produce two different types of melanin: brown-black eumelanins and yellow-red pheomelanins. Both eumelanins and pheomelanins are formed through a series of transformations of DOPAquinone, a product of the oxidation of the amino acid tyrosine.

Eumelanins constitute a group of insoluble pigments composed of subunits including 5,6-dihydroxyindole (DHI), 5,6-dihydroxyindole-2-carboxylic acid (DHICA), and pyrrolic subunits derived from the peroxidative breakdown of DHI and DHICA. Eumelanins are composed of carbon, hydrogen, oxygen, and nitrogen atoms (6–9%) [26,27].

Pheomelanins are primarily composed of benzothiazine subunits and, to a lesser extent, benzothiazole and isoquinoline subunits [27]. It is important to emphasize that isoquinoline subunits are likely formed as a result of post-polymerization modifications, as they are not produced during earlier stages of pheomelanogenesis. Unlike eumelanin, pheomelanins dissolve in alkalis and contain sulfur atoms (9–12%) in their structure, originating from sulfhydryl compounds such as cysteine [26].

In addition to the types of melanin mentioned above, mammals also have a category known as neuromelanins. They are found in the central nervous system, primarily in the substantia nigra and locus coeruleus [28]. Neuromelanins are brown-black pigments that consist of a mixture of eumelanin and pheomelanin [29]. Their synthesis occurs in dopaminergic and noradrenergic neurons as a result of the metabolism of catecholamines that have not been stored in synaptic vesicles. The structure of neuromelanin includes dihydroxyindole and benzothiazine subunits [28]. In addition to the pigment itself, neuromelanin also contains a protein component (15% of its mass) and a lipid component, with its main constituents being dolichol and long-chain saturated and unsaturated fatty acids [30].

**Figure 2:**
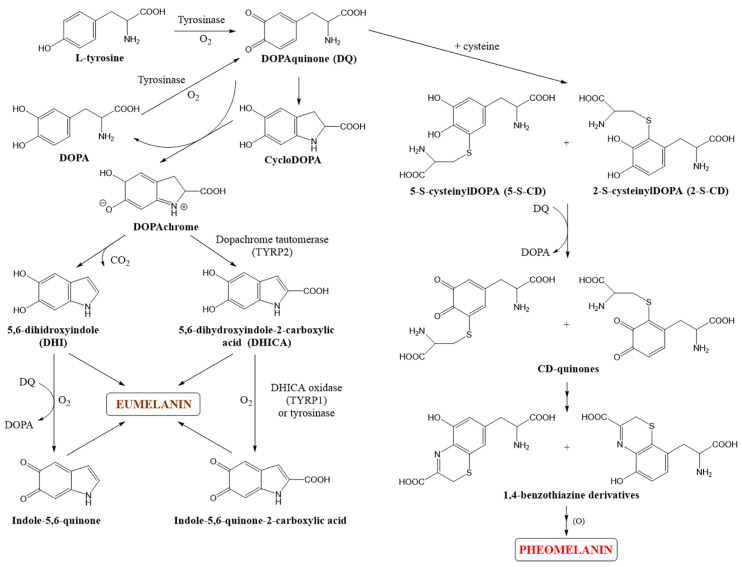
The scheme of biosynthesis of melanin polymers.

Melanogenesis is a complex and multi-stage process leading to the production of melanin pigments (Figure 2). The biosynthesis process of melanin, known as the Raper–Mason–Prota pathway, begins with the oxidation of L-tyrosine to DOPAquinone [27]. This reaction is catalyzed by tyrosinase, a key enzyme in melanogenesis. Further transformations of DOPAquinone depend on the presence and concentration of thiol compounds, such as cysteine or glutathione, inside melanosomes. It has been shown that if the cysteine concentration is higher than 0.13 µM, it reacts with DOPAquinone (DQ), resulting in the formation of 2-S- and 5-S-cysteinylDOPA (CD), redirecting melanogenesis towards pheomelanin synthesis. In the next step, a redox reaction occurs between CD isomers and DQ, leading to the formation of CD-quinones and DOPA (3,4-dihydroxyphenylalanine). CD-quinones subsequently undergo cyclization and rearrangement into 1,4-benzothiazine derivatives, which, through oxidation and polymerization, give rise to yellow-red pheomelanin [27,31].

When the cysteine concentration is lower than 0.13 µM, DOPAquinone undergoes intramolecular cyclization to form cycloDOPA. This compound is then oxidized in a redox reaction with DQ to form DOPAchrome. In this reaction, DQ is reduced to DOPA, which can be oxidized back to DQ by tyrosinase. Orange-red DOPAchrome further undergoes non-enzymatic decarboxylation to form 5,6-dihydroxyindole (DHI) or tautomerization to form 5,6-dihydroxyindole-2-carboxylic acid (DHICA) under the influence of TYRP2. Hydroxylated indole derivatives are then oxidized to indole-5,6-quinone and indole-5,6-quinone-2-carboxylic acid by TYRP1 (e.g., in mice) or tyrosinase (in humans) [32,33]. It is worth emphasizing that human tyrosinase, which also exhibits DHICA oxidase activity, unlike human TYRP1, has a broader substrate specificity compared to mouse tyrosinase. In the final step, these quinone compounds polymerize to form brown-black eumelanin [27,31,34].

In vivo melanogenesis results in the production of a mixture of pheomelanin and eumelanin. Therefore, we actually have a “mixed melanogenesis” process consisting of three main consecutive stages. The first stage involves the synthesis of cysteinylDOPA isomers, which continues as long as the cysteine concentration remains above 0.13 µM. The second stage includes the oxidation of CD isomers and pheomelanin production, which continues as long as the CD concentration remains above 9 µM. The third and final stage of “mixed melanogenesis” is the synthesis of eumelanin. This sequence of stages is reflected in the model of melanin complexes, where the pheomelanin core is covered by a layer of eumelanin [31,35].

The regulation of melanogenesis is complex and involves the participation of physical factors such as UV radiation, as well as biochemical factors including endocrine, paracrine, and autocrine factors. Many of the factors that stimulate melanogenesis originate from the keratinocytes surrounding melanocytes. These factors include basic fibroblast growth factor (bFGF), stem cell factor (SCF), hepatocyte growth factor (HGF), granulocyte-macrophage colony-stimulating factor (GM-CSF), nerve growth factor (NGF), melanocyte-stimulating hormone alpha (α-MSH), adrenocorticotropic hormone (ACTH), endothelin 1, prostaglandin E2 and F2α. Some of these factors, such as bFGF, SCF, and HGF, are also secreted by fibroblasts, while α-MSH and ACTH are hormones primarily produced in the pituitary gland. Other endogenous factors that can stimulate melanogenesis include estrogens, nitric oxide, histamine, endorphins, thromboxane B2, and leukotrienes C4 and D4. On the other hand, androgens, interleukin 1 and 6, tumor necrosis factor-alpha (TNF-α), sphingolipids, and bone morphogenetic protein 4 (BMP-4) have been shown to have inhibitory effects on melanin biosynthesis [36,37].

All of the above-mentioned physiological factors influence the activity of various signaling cascades that regulate melanogenesis. The most important cascade for melanin biosynthesis is the signaling pathway associated with the melanocortin receptor MC1-R (melanocortin receptor 1). Stimulation of the MC1-R receptor by an agonist such as α-MSH or ACTH leads to the activation of adenylate cyclase and an increase in intracellular cAMP (cyclic adenosine monophosphate) levels, which is a significant stimulator of melanogenesis. cAMP, through the activation of protein kinase A, ultimately induces the expression of the transcription factor MITF—the master regulator of melanogenesis at the transcriptional level. MITF induces the expression of genes responsible for the biogenesis, maturation, and transport of melanosomes, as well as the course of melanin biosynthesis, including genes encoding tyrosinase, TYRP1, and TYRP2 [38,39].

The most important exogenous factor regulating melanogenesis in humans is UV radiation. It is the primary stimulator of the induced skin pigmentation process known as tanning [40]. Melanin production occurs to a much greater extent in the skin or in co-cultures of melanocytes and keratinocytes compared to isolated melanocytes, due to the involvement of keratinocytes in stimulating melanogenesis under the influence of UV radiation [41].

The molecular and biochemical aspects of UV radiation-induced melanogenesis are not fully understood at present. It has been observed that under the influence of UV radiation, proteins such as p53, protein kinase C beta (PKC-β), and the ion channel transient receptor potential A1 (TRPA-1) are activated, among other factors [37,40,42].

## 5. Roles of Melanin

Melanins are among the most important factors determining the phenotypes of organisms. The color of skin, hair, and eyes is primarily determined by melanin pigments. It is worth noting that differences in pigmentation between different populations do not result from the number of melanocytes but from the amount and type of melanin synthesized, as well as the shape and distribution of melanosomes. The number of melanocytes itself does not change throughout one’s life, but the density of their distribution in different parts of the skin varies, ranging from approximately 900 melanocytes/mm^2^ on the back to around 1500 melanocytes/mm^2^ in the genital areas [34]. Lightly pigmented skin contains a mixture of eu- and pheomelanin, with smaller, less pigmented melanosomes grouped together. In contrast, darkly pigmented skin mainly contains eumelanin, with larger, more pigmented melanosomes that are distributed individually [43]. Considering skin pigmentation and response to sunlight exposure, a scale of skin phototypes was developed by American dermatologist Thomas B. Fitzpatrick. This scale distinguishes six different phototypes, as presented in Table 1. Skin phototypes are classified according to the Fitzpatrick scale, which is based on skin and eye color, as well as susceptibility to sunburn and tanning under UV radiation [38,44,45]. Individuals with low skin phototype values have very fair skin, a low number of melanosomes, and primarily synthesize pheomelanin. On the other hand, the skin of people with a high phototype has numerous melanosomes filled mainly with eumelanin. In other cases, eumelanin is synthesized in small quantities, but the number of melanosomes is substantial.

One of the most important functions of melanin is to protect cells from the harmful effects of UV radiation and eliminate free radicals, primarily reactive oxygen species, such as singlet oxygen, hydrogen peroxide, and superoxide anion. Both factors can cause damage to the structure of nucleic acids (DNA and RNA), proteins, and lipids, leading to the loss of their function and disturbances in cell physiology. UV radiation reaching the Earth’s surface primarily consists of UVA radiation (320–400 nm) and, to about 5%, UVB radiation (280–320 nm). UVC radiation (200–280 nm) does not reach the Earth’s surface as it is absorbed by the ozone layer. The effects of UV radiation on the skin can be categorized into short-term effects such as erythema, mutations, immunosuppression, vitamin D synthesis, and tanning, as well as long-term effects like photoaging and photocarcinogenesis [46].

Skin pigmentation serves as the primary defense mechanism against the adverse effects of UV radiation. The protective role of melanin is due to its ability to scatter and absorb UV radiation. The absorbed energy can be converted into less toxic thermal energy. Melanin’s ability to absorb radiation is highest in the short-wavelength UV range and gradually decreases as it transitions towards visible light [36]. It is estimated that melanins can absorb 50–75% of UV radiation [37,46]. From a cellular perspective, the most important role of melanin is to protect genetic material. Therefore, melanosomes located in keratinocytes form protective caps over the cell nuclei, preventing DNA damage from UV radiation. The photoprotective role of melanin has been confirmed by numerous epidemiological studies, which indicate an inverse relationship between the incidence of skin cancers, including melanoma, and the degree of pigmentation [47]. However, it should be noted that pheomelanin, unlike eumelanin, undergoes photodegradation under the influence of UV radiation, resulting in the production of hydrogen peroxide, superoxide anions, and potentially causing mutations in melanocytes and other cells [46]. Additionally, intermediate products of melanin synthesis are considered toxic and may participate in generating free radicals under the influence of UV radiation, leading to DNA damage [37,48]. Furthermore, it has been found that pheomelanin increases the risk of apoptosis in cells exposed to UV radiation and enhances the release of histamine, which contributes to the development of erythema and edema—clinical signs of phototoxicity [46]. The low photostability of pheomelanins does not offer effective photoprotection for cells and may also increase the risk of carcinogenesis. It has been found that individuals with fair skin are 70 times more likely to develop skin cancer than those with dark skin. It is worth noting that one of the causes of pheomelanin-induced tumorigenesis is the low intracellular level of the antioxidant glutathione, which serves as the source of cysteine used during pheomelanin synthesis [49].

Regardless of the type of melanin synthesized, the process of melanogenesis itself may be harmful to pigment cells. The synthesis of melanin biopolymers, despite being a defense mechanism, involves an oxidative process that generates highly reactive intermediates with cytotoxic and genotoxic properties. Additionally, the stimulation of glycolysis and activation of hypoxia-inducible factor 1-alpha observed during melanogenesis may contribute to melanoma progression and increased resistance to immunotherapy. Therefore, inhibiting melanin synthesis could be a promising target for new adjuvant therapies in the case of melanoma [50].

## 6. Interaction of Drugs with Melanins

Biopolymeric melanins have the ability to form complexes with many chemical substances, including drugs, affecting their therapeutic efficacy, toxicity, and the occurrence of side effects.

On one hand, a drug bound to pigments loses its pharmacodynamic properties, while on the other hand, it accumulates in pigmented cells, increasing the risk of damage to these cells and the occurrence of side effects. This particularly applies to phototoxic effects and toxicity towards the eyes and ears [51,52]. Taking into account the pharmacological aspect of drug–melanin complex formation, it is necessary to consider the need for higher drug doses to achieve the therapeutic concentration of the free fraction of the drug. This is due to the fact that drug molecules bound to melanin biopolymers cannot interact with the appropriate receptors. At the same time, drugs accumulated in pigmented tissues serve as a kind of “depot” form, extending the exposure time of cells to their effects and influencing the process of their local biodistribution [53,54].

The binding of chemical substances depends on their structure and physicochemical properties, such as their acid-base character and lipophilicity. It has been found that all drugs binding to melanins have basic groups. Similarly, most drugs forming complexes with melanins are lipophilic substances with a positive logP value (octanol/water partition coefficient) [51]. The presence of various functional groups in the structure of melanin, such as carboxyl, hydroxyl, phenolic, amino, and imino groups, gives them the ability to form complex compounds with metal ions, alkaloids, dyes, or herbicides [52]. The polyanionic nature of melanin allows it to bind substances with a positive charge by forming ionic bonds. Hydrophobic interactions, van der Waals forces, and “charge transfer reactions” also play an important role in the formation of melanin complexes with chemical substances [51,52]. The formation of drug–melanin complexes is a specific process and does not correlate with the interaction of drugs with other macromolecules, including blood proteins [55]. It should be emphasized that the binding parameters for individual drugs may even differ between synthetic and biological melanin. This is influenced by different physicochemical properties, the shape and size of molecules, and the specific surface area of melanin. For this reason, melanin polymers of biological origin are considered the most reliable model for studying drug binding [53,56,57].

Melanin-binding drugs include, but are not limited to, aminoglycoside antibiotics (tobramycin, neomycin, and amikacin) [58], tetracyclines (doxycycline and chlortetracycline) [59,60], fluoroquinolones (ciprofloxacin and lomefloxacin) [61], antifungal drugs (amphotericin B and caspofungin) [62], psychotropic drugs (chlorpromazine, fluphenazine, and trifluoperazine) [63], local anesthetics (tetracaine, procaine, bupivacaine, and lidocaine) [64], non-steroidal anti-inflammatory drugs (ketoprofen) [65], anticancer drugs (daunorubicin and doxorubicin) [66], and antimalarial drugs (chloroquine and hydroxychloroquine) [67]. Drugs that are significantly complexed by melanin include tropane alkaloids (atropine), β-adrenergic blockers (betaxolol and carteolol), α-adrenomimetic drugs (tizanidine), and β-lactam antibiotics (penicillin G and ampicillin) [55].

Many of the aforementioned drugs can induce cutaneous side effects, including phototoxic reactions and pigmentary disturbances. It appears that the formation of drug–melanin complexes and drug accumulation in the skin may exacerbate the risk of such effects. The phototoxic properties of selected drugs binding to melanin have been demonstrated, including in in vitro studies on normal epidermal melanocytes. It has been shown that even a relatively low dose of UVA radiation (1.3 J/cm^2^) intensifies the toxic effect of tetracyclines and fluoroquinolones in darkly pigmented melanocytes cultured with the drug for 24 h before irradiation [68,69,70]. This indicates that the presence of photoprotective melanin may not protect against the phototoxicity of drugs accumulated in melanocytes. It is worth noting that this may be partly due to the limitation of the antioxidant capacity of melanin by the bound drugs [71].

Adverse effects associated with drug–melanin complexation are not limited to the skin. It has been demonstrated that drug binding by the retinal pigment epithelium melanin contributes to drug retention in the eye. Drug accumulation in the eye has been confirmed for various drugs, including levofloxacin, terazosin, papaverine, and timolol [72]. It has also been shown that the chemisorption of chloroquine on the surface of melanin biopolymers contributes to drug-induced retinopathy [71]. Evidence includes a weaker interaction of hydroxychloroquine with melanins. It has been shown that the hydroxy derivative of chloroquine binds to melanins to a much lesser extent than the parent compound, making it a safer drug in terms of harmful effects on the eyes [67,71]. However, it should be emphasized that analyses of drug–melanin binding parameters did not ultimately yield positive results in predicting drug-induced ototoxicity [73]. This may be partly due to the fact that the pharmacokinetic profile of drugs in the eye results not only from the drug–melanin interaction but also from various physicochemical properties, cell permeability, and binding to plasma proteins [74].

Moreover, drugs penetrating the central nervous system, such as phenothiazine derivatives, haloperidol, or imipramine, may bind to neuromelanin in substantia nigra neurons, contributing to their degradation and the occurrence of dyskinesias [75]. It has also been found that the chelating properties of melanin lead to the accumulation of metal ions in the central nervous system, which may underlie the development of Parkinson’s disease [76]. It is presumed that melanins, by binding metals, drugs, or toxins, provide protective effects only in the initial stage. The reversibility of substance binding to melanins means that over time, they start to be released into the cytosol, leading to cell damage and the occurrence of adverse or toxic effects, including neurodegenerative ones [77,78].

It is worth noting that the formation of complexes between drugs and melanin is currently being utilized to create new drug delivery systems. In this case, melanin nanoparticles are treated as carriers for controlled drug release. Model studies have been conducted with chloroquine, propranolol, timolol, and diclofenac, among others [79]. Melanin, as a drug carrier, possesses many valuable properties. It is a biocompatible and biodegradable material with a large surface area and binding capacity, as well as adhesive capabilities. Additionally, it acts as an antioxidant, exhibits chelating properties, is photoconductive, and has the ability for photo-thermal conversion. All these features make melanin nanoparticles a promising material used in photodynamic therapy, photothermal tumor therapy, specific multimodal imaging, photoacoustic imaging, and as theranostics—substances combining targeted therapy and diagnostic testing [80,81,82,83]. Due to melanin’s ability to directly scavenge free radicals, attempts are being made to use melanin nanoparticles to protect against the harmful effects of X-ray radiation on skin cells, facilitated by the effective penetration of melanin nanoparticles into keratinocytes [84].

## 7. Skin Pigmentation Disorders—Definition and Classification

As outlined above, the color of human skin depends mainly on pigment content. The primary pigments affecting skin color include the biopolymers melanin, carotene, and reduced and oxygenated hemoglobin [85]. Other factors influencing skin color and appearance include the thickness of the epidermal layer and the number and reactivity of blood vessels located in the dermis [86]. The synthesis of melanin biopolymers is regulated by genetic factors, UV exposure, biochemical substances, and hormonal influences. Factors involved in the regulation of the melanization process are responsible for the proper formation, regeneration, heterogeneity, and aging of melanocytes and their precursors. The main elements involved in the melanization process are proteins necessary for the correct construction of melanosomes, enzymes involved in the multi-step process of melanin synthesis, and proteins involved in the distribution of the synthesized pigment. More than 4000 different skin diseases are currently known, many of which are associated with pigmentation disorders [87]. Currently known animal models allow us to conclude that nearly 350 genetic loci are responsible for the normal pigmentation process. Many of the genes whose mutations are responsible for pigmentation disorders are responsible for the complexity of the production, transport, and regulation of melanin biopolymers [88].

Pigmentation abnormalities comprise a large group of heterogeneous disorders that may be caused by a number of factors, such as abnormal migration of melanocytes from the neural crest to the dermal layers during embryogenesis, abnormal melanosome transfer to keratinocytes, abnormal melanogenesis and impaired melanin concentration, melanocyte density, or melanocyte degradation [89,90]. Melanin abnormalities are divided into two types: hypopigmentation or hyperpigmentation, which can be congenital or acquired [91].

## 8. Hyperpigmentation

Hyperpigmentation is a group of diseases that include both congenital forms, with different patterns of inheritance, and acquired forms caused by skin or systemic problems. The vast majority of them are a result of excess melanin synthesis, distribution, or transport and increased tyrosinase activity [92]. Additionally, this defect may be connected with increased lipofuscin production, inflammation occurrence, or the deposition of chemical compounds in the skin tissues. Moreover, about 10–20% of hyperpigmentation disorders are an adverse effect of pharmacotherapy [93].

### 8.1. Congenital Hyperpigmentation

Congenital hyperpigmentation includes pigmentation disorders specific to the epidermis, which include Spitz nevi, Spilus nevi, and cellular nevi, and within the dermis, which include Ohta nevi, dermal hyperpigmentation, Ito nevi, and blue nevi. In addition, there are congenital hyperpigmentations such as LEOPARD syndrome, generalized lentiginosis, Carney syndrome, Cronkhite–Canada syndrome, and Peutz–Jeghers syndrome [87].

Generalized lentiginosis (GL), the occurrence of which is associated with a mutation in a gene located on chromosome 4q21.1-q22.3, results in the presence of a particularly large number of lentiginous spots characteristically distributed on mucous membranes and skin [87,94]. Lentiginosis includes forms that are strictly confined to a single site or are widespread and may be part of other syndromes. In GL, multiple lentiginous patches are found that do not have a characteristic distribution and are not accompanied by other systemic disorders. Most lentiginous spots are associated with a variety of cardiovascular, neurological, gastrointestinal, musculoskeletal, and auditory disorders, which are classified as separate subunits—LEOPARD syndrome and Carney syndrome [93]. LEOPARD syndrome (LS) is a sporadic missense mutation in protein-tyrosine phosphatase type 11(PTPN11). Hyperpigmentation is inherited in an autosomal dominant manner, accompanied by clinical abnormalities such as lentigines spots, and congenital disorders of the cardiac, ocular, pulmonary, reproductive, auditory, and deafness systems. Due to its rarity and difficult diagnosis, LS is a poorly described disease entity [95]. Carney syndrome (CNC) is a rare hyperpigmentation disorder inherited as an autosomal dominant trait. A genetic mutation in the regulatory subunit of protein kinase A 1α (PRKAR1A) is present in almost 70% of CNC cases [15]. Carney syndrome is associated with pigmented lesions of the skin and mucous membranes, mucinous tumors of the skin and heart, and non-endocrine and endocrine neoplasms [96]. Peutz–Jeghers syndrome (PJS) is associated with mutations in the STK11 (serine/threonine kinase)/LKB1 gene inherited in an autosomal dominant manner. Clinical manifestations of PJS include the presence of polyps in the gastrointestinal tract and mucocutaneous discoloration on the lips. PJS has a significantly higher susceptibility to the development of malignancies of multiple organs, i.e., the lung, breast, and genitourinary tract, compared to the general population [97]. Laugier–Hunziker syndrome is an inherited hyperpigmentation disorder characterized by the expression of pigmentation on nails, mucous membranes, and acral areas. Although the disease is known to have a benign course, there have been several reports linking it to esophageal melanocytosis, hypocellular bone marrow, lichen planus, and thrombocytopenia [98]. The most likely mechanism for Laugier–Hunziker syndrome is the presence of large L-3,4-dihydroxyphenylalanine dendritic melanocytes in the epidermis, which are characterized by increased melanogenesis [99]. One of the most commonly diagnosed pigmented lesions in Asian and negroid neonates is the Mongolian spot, classified as cutaneous melanocytosis. The grayish blue areas are mainly located in the sacrococcygeal and lumbar regions. This type of hyperpigmentation usually disappears by the age of 1–6 years and does not require special treatment due to its benign course [100]. Lentigine spots are associated with an increased number of melanocytes and melanin production. They are genetically determined and have been present since childhood. Lentigines spots can be located all over the body and on mucous membranes. Unlike freckles, lentiginous spots are larger in size, darker in color and more oval in shape [101]. Freckles, lentiginous spots, and periorbital hyperpigmentation are types of congenital hyperpigmentation disorders inherited in an autosomal dominant manner. Freckles are small, smooth, light or dark brown patches located around the exposed body—mainly on the face, limbs, and back—which are characterized by a localized increase in melanin pigment production. The color of freckles can change throughout the year, depending on the intensity of sunlight. Light complexions and red hair are predisposing factors for a higher incidence of freckles [102].

### 8.2. Acquired Hyperpigmentations

One acquired hyperpigmentation disorder is melasma, characterized by an increased number of melanosomes and levels of hypertrophied melanocytes. In addition, there is increased melanin deposition in all layers of the epidermis [103]. Melasma is associated with asymmetric, irregular brown patches and a macular appearance. The location is predominantly on the face, but it can also occur less frequently on the arms, chest, or neck [104]. Currently available global epidemiological data indicate a large population variation in the prevalence of melasma. A significantly higher incidence of melasma has been reported in regions with particularly high rates of UV irradiation, such as Latin America, Asia, and Africa [105]. Although the pathogenesis of melasma formation has not yet been fully elucidated, frequent UV exposure, genetic predisposition, and female hormonal stimulation are known to play a key role in the development of melasma, predisposing women to develop melasma more frequently (approximately 90% of cases) [92,106]. Hyperactivation of the estrogen receptors of b-type melanocytes (ERb) triggers melanin production by increasing the transcription factors tyrosinase gene PDZK1 and melanocortin receptor type 1 (MC1R) expression [107,108]. Other skin cells involved in melasma development, such as dermal fibroblasts or epidermal keratinocytes, may modulate the Wnt signaling pathway. Reduced expression of WIF-1 (Wnt Inhibitory Factor 1) stimulates melanosome transfer and melanin synthesis through up-regulation of the Wnt pathway [106]. Additionally, UV radiation stimulates melanogenesis through the release of histamine from mast cells, which affects H2 receptors and activates kinase A [109]. Riehl’s melanosis (RM) is commonly classified as pigmentary contact dermatitis (PCD). RM is an acquired hyperpigmentation caused by low-grade allergic contact dermatitis to ingredients in cosmetic products, such as fragrances. Abnormal hyperpigmentation occurs mainly on the face and neck and mainly affects Asian patients [110]. Post-inflammatory hyperpigmentation (PIH) is an acquired hyperpigmentation that can affect all skin types, but is more common in darker-skinned individuals compared to Caucasians due to more intense melanin pigment biosynthesis [111]. Endogenous and exogenous factors are involved in the development of PIH. Common endogenous factors causing PIH are psoriasis, acne vulgaris, rosacea, eczema, or atopic dermatitis. External factors may be related to skin burns, allergies, insect bites, radiotherapy, or dermatological therapies such as laser treatments or chemical peels [112]. The primary cause of PIH is inflammation, which leads to the overproduction of melanin and/or irregular pigment dispersion. Inflammatory mediators such as interleukins (ILs): IL-1, IL-6; leukotrienes (LTs): LT-C4 and LT-D4; prostaglandins E2 and D2; thromboxane-2; tumor necrosis factor (TNF)-α; epidermal growth factor; and reactive oxygen species (ROS) have all shown melanogenesis-stimulating properties [113]. Two main histopathological types of post-inflammatory hyperpigmentation can be observed. The first type is associated with increased melanin transfer to surrounding keratinocytes, when hyperpigmentation is confined to the epidermis, taking on the appearance of brown or dark brown patches [111]. The second mechanism involves PIH in the dermis and results from inflammation-induced damage to basal keratinocytes, which release melanin. Free melanin pigment is phagocytized by macrophages, which transform into melanophages. Melanophages are located in the upper dermis and cause a blue-gray appearance of the skin at the site of injury [112]. PIH hyperpigmentation disorders usually occur around sun-exposed areas, such as the face, forearms, and back [113]. PIH can persist for up to several months after the removal of the inflammatory agent [114]. Solar hyperpigmentation disorders are characterized by dark spots that appear on sun-exposed skin areas such as the face and arms, similar to PIH. Due to natural defense mechanisms, human skin has the ability to protect itself from UV radiation. One mechanism is related to the thickening of the epidermis; the other, the stimulation of melanin synthesis, is more important in photoprotection. It is known that melanin pigment protects the skin from UV damage by blocking it and dissipating it as harmless heat. As a result of UV exposure, melanin pigment darkens and its production increases, causing the appearance of localized hyperpigmented lesions [87,115,116]. An acquired hyperpigmentation observed mainly in the elderly population is lentigo sensilis. Old-age lentiginous spots are characterized by a diameter of approximately 1 cm and a beige or brown color. Lentiginous spots appear on parts of the body exposed to UV radiation, such as the face, décolleté, hands, and arms, and are associated with photodamage. Currently available data suggest a potential mechanism for the formation of senile spots as a result of paracrine stimulation of melanogenesis by numerous factors, including hepatocyte growth factor (HGF), endothelin 1 (EDN1), keratinocyte growth factor (KGF/FGF7), and stem cell factor (SCF/KITL). Because senile lentigines increase in number and size with age, they require dermatological follow-up, as skin cancers such as melanoma can originate from lentigines [101]. Cronkhike–Canada syndrome is a very rare disease, with approximately 450 cases described to date. The disease is characterized by multiple gastrointestinal polyps, weight loss, skin discoloration, alopecia, and dystrophic nail changes. Currently, the etiology of the disease is not fully known, but due to its rarity, it is not considered an inherited disease. Cronkhike–Canada syndrome is most likely an autoimmune disease associated with inflammation and elevated IgG4 levels. The symptoms associated with the disease, including gastrointestinal bleeding, anemia, and malnutrition, result in a high mortality rate, estimated at more than 50% of diagnosed cases [117,118]. Hyperpigmentation disorders are also observed as one of the symptoms in the course of other systemic diseases such as mastocytosis, which is a rare childhood disease associated with abnormal expansion and the deposition of mast cells in the various organs that include the spleen, gastrointestinal tract, lymph nodes, and skin [119]. Mastocytosis is classified into advanced and non-advanced types. The non-advance type characterized by a good prognosis includes patients with a cutaneous type of disease where the skin is the only affected organ. The cutaneous components of mastocytosis are associated with the presence of neoplastic mast cells in the papillary layer of the dermis, manifesting as yellow or red-brown spots up to 1 cm in diameter, present on the range and trunk. Conversely, in the case of advanced mastocytosis, mast cell leukemia, hematological malignancy, and a poor prognosis are observed [120]. Neurofibromatosis type 1 (NF1) is an autosomal dominant disease that manifests as multiple flat, brown patches resembling café-au-lait spots, subcutaneous neurofibromas, and Lisch nodules. Currently, 3000–4000 cases have been reported worldwide, and patients have a reduced life expectancy by an average of 15 years compared to the general population, which is associated with an increased risk of developing malignant tumors and vascular diseases in the course of NF1 [121]. Café-au-lait spots are also observed as a cutaneous manifestation of the symptoms of McCune–Albright syndrome. Discolorations on the midline of the body appear immediately after birth and take on a characteristic irregular jagged appearance [122]. Moreover, skin pigmentation disorders are observed in the course of many rare diseases, including POEMS syndrome, Sotos syndrome, and Cantu syndrome, as well as many systemic diseases related to impaired functioning of the endocrine system, liver, and kidney diseases, as well as metabolic and infectious diseases [87].

## 9. Hypopigmentation

Hypopigmentation disorders can be divided into melanoses connected with abnormal melanocyte function or melanocytoses connected with melanocyte development. These disorders may appear in the form of hypomelanocytosis or hypomelanosis. A complete or partial absence of melanocytes is a disorder that causes hypomelanocytosis. Among the mechanisms underlying hypopigmentation disorders, the following disorders can be distinguished [90]:

- Disturbances in the migration of melanoblasts from the neuroectoderm to the skin, which cause diseases such as piebaldism and Waardenburg syndrome.
- Diseases caused by abnormal melanogenesis, e.g., vitiligo and Menkes syndrome.
- Abnormal formation of melanosomes that may cause Hermansky–Pudlak (HPS) disease and Chediak–Higashi syndrome (CHS).
- Abnormalities in melanosome transfer are the cause of Griscelli syndrome (GS).

Among the hereditary hypomelanocytosis, Waardenburg syndrome, piebaldism, oculocutaneous albinism, Chediak–Higashi syndrome, Griscelli syndrome, and Tietz syndrome can be distinguished. Vitiligo is another type of hypopigmentation disorder caused by an autoimmune system disease [123].

### 9.1. Congenital Hypomelanoses

Albinism is a genetic defect inherited in an autosomal recessive manner that is the cause of radical reduction or complete absence of melanin, with an incidence estimated at 1:5000–1:40,000 cases [90]. Abnormal functioning of the tyrosinase enzyme is the cause of the symptoms of albinism, which include a pale complexion, fair or white hair, and light blue eyes. Limiting the protective role of melanin makes albinos highly susceptible to the adverse effects caused by UV radiation, which is associated with a great risk of skin cancer development, e.g., squamous cell carcinoma or basal cell carcinoma [58]. Moreover, syndromic and non-syndromic forms of albinism can be distinguished. Chediak–Higashi (CHS) and Hermansky–Pudlak (HPS) subtypes are syndromic albinism forms in which, besides hypopigmentation disorders, other pathological abnormalities coexist. Lysosomal trafficking regulator (*LYST*) gene mutations underlie CHS disease, the main symptoms of which are partial oculocutaneous albinism, immune dysfunction, neurodegeneration, prolonged bleeding, and a risk for hemophagocytic lymphohistiocytosis development [124,125]. The defective biogenesis of multiple tissue-specific lysosome-related organelles (LROs), including melanosomes, is characteristic of HPS, whose typical symptoms are pulmonary fibrosis, immunodeficiency, high bleeding tendency, and neuropsychological disorders [126]. Waardenburg syndrome is a rare auditory–pigmentary disease that is inherited in an autosomal dominant manner. It is characterized by local loss of melanocytes in the skin, eyes, and hair. The characteristic features of Waardenburg syndrome are also pigmentary abnormalities, hypertrichosis of the medial part of the eyebrows, white forelock, dystopia canthorum, and broad nasal root [127]. This disease is responsible for 2–3% of congenital deafness related to the loss of melanocytes in the cochlear stria vascularis. The estimated prevalence of the disease accounts for 2–3 cases per 100,000 people. The basis for the occurrence of the Waardenburg syndrome is gene mutations, which can include deletion, insertion, missense, and nonsense mutations [128]. Four clinical variants of this hypopigmentation can be identified; however, types 1 and 2 are the most common. The mutation in the *PAX3* gene is responsible for the occurrence of syndrome type 1, which manifests by the presence of irregular depigmentation areas located on the limbs, abdomen, and chest, white eyebrows, eyelashes, deafness, and irises heterochromia. In addition, face and hand malformations can also be present [57]. The second type of Waardenburg syndrome is connected with *MITF* gene mutations, which result in the replacement of Ser^298^ with alanine or proline. The symptoms of Waardenburg syndrome type 2 are similar to those of type 1, but they are less severe. Type 3 Waardenburg syndrome is inherited autosomal recessive or dominant and may be caused by mutations in the *PAX3* gene [129]. This type of disease is characterized by pigmentation disorders such as polyposis, iris heterochromia, deafness, abnormalities in the musculoskeletal system, respiration disorders, heart defects, and dystopia canthorum. It is postulated that some types of mutations accompanying type 3 Waardenburg syndrome may cause a much more severe course of the disease, leading to death in early childhood [130]. Type 4 Waardenburg syndrome is associated with mutations within the endothelin genes. An additional symptom accompanying type 4 disease is, despite depigmentation changes, a disturbance in the proper functioning of the intestines associated with the deficiency of neurons in the intestinal plexus [131]. Tietz syndrome is a rare inherited disorder associated with the *MITF* gene mutation in which the replacement of Lys^210^ with asparagine and the deletion of Arg^217^ are observed. This disease is characterized by hypopigmentation disorders, including general depigmentation, blue eyes, blonde hair, eyelashes, and eyebrows, and complete deafness. During solar radiation, reddish freckles can occur [123]. Griscelli syndrome (GS) is an exceptionally occurring disease inherited in an autosomal recessive manner. Characteristic hypopigmentation of hair (silver-gray color) and skin caused by incorrect melanosome transfer to keratinocytes coexisted with neurological or immunological disorders, allowing to distinguish three subtypes of GS [90,132]. The distinguished types of Griscelli syndrome differ in terms of hypopigmentation changes. In GS type 1, the clinical symptom is complete hypopigmentation, while type 2 is characterized by only partial hypopigmentation changes and a lack of hair color. The effects of Griscelli syndrome type 3 are the result of hypopigmentation changes in the skin; they do not cause changes in other organs. Type 1 Griscelli syndrome is a mutation of the myosin gene *MYO5A*, which is located specifically on chromosome 15q21 of the true missing transporter of melanocytes. In the case of GS type 2, the mutation is in the *RAB27A* gene located on chromosome 15q21. At the cellular level, Griscelli syndrome shows the accumulation of mature melanosomes in the perinuclear region. GS type 3 represents a limited expression of the disease and is characterized by hypopigmentation in hair and skin, and it is caused by a mutation in the gene located on chromosome 2q37.3, which encodes melanophilin, which is an effective member of the *RAB* family. Piebaldism is a rare autosomal dominant disorder characterized by a congenital lack of pigment cells in the scalp, torso, and limb areas. The estimated prevalence of piebaldism accounts for 1 case per 100,000 births, in both men and women. The local lack of melanocytes is caused by abnormal melanoblast proliferation, migration, and survival during the embryogenesis process [133]. These disorders result from a reduction in the number of KIT receptors on melanoblasts’ surfaces, which disrupts the melanoblast’s development and inhibits melanogenesis activity. White skin and hair spots are characteristic of patients with piebaldism, but paradoxically, hyperpigmentation disorders may also appear during the course of this disease [123,134].

A summary of the hereditary pigmentary diseases presented in this manuscript is shown in Table 2.

### 9.2. Acquired Hypomelanoses

Idiopathic guttate hypomelanosis (IGH) is an acquired hypopigmentation that affects all skin types and races and is characterized by numerous oval, small, discrete, porcelain-white macules localized mainly to sun-exposed areas such as the forearms. IGH affects people in their 40s and develops slowly over many years [135]. The available data show that IGH is present in approximately 80% of the population by the age of 70. The multifactorial pathogenesis of IGH, combining both environmental and genetic conditions such as UV exposure, autoimmunity, aging, trauma, or local inhibition of melanogenesis, results in a large number of diagnosed cases [136]. IGH is an effect of reducing the total number of melanocytes, but other opinions suggest structural abnormalities of melanocytes, such as reduced tyrosinase activity, a reduction in the number of melanosomes, or adjacent keratinocyte defects [137]. One of the rare side effects of both UVA and UVB phototherapy in patients with psoriasis is leukoderma punctata. Typical clinical features of leukoderma punctata are multiple round lesions with sharp edges, often on the upper chest and back and occasionally on the face [138,139]. This condition usually occurs in fair-skinned females before the age of 40, in contrast to IGH. It should be emphasized that leukoderma punctata may also be a delayed adverse effect of Q-switched and carbon dioxide lasers. Multifunctional hypopigmentation disorder—vitiligo is the most common skin depigmentation disorder, affecting on average 0.5–2% of the total population. Vitiligo is characterized by the local loss of functional melanocytes, resulting in pigment dilution and the appearance of white patchy depigmentation [140]. Vitiligo is an autoimmune disease that results in the progressive destruction of skin melanocytes by multiple mechanisms. Approximately 80% of the risk of developing vitiligo is related to genetic factors, and the remaining 20% is attributed to oxidative stress, autoimmune responses, inflammation, and melanocyte dysfunction [141]. Based on epidemiological studies, it could be concluded that vitiligo tends to occur in families, which represent 9% of all cases [31]. Large-scale genome-wide association studies have identified about 50 different genetic loci that confer risk for vitiligo [142]. Many of the identified vitiligo loci are involved in melanin pigment synthesis, immune regulation, or apoptosis and are also associated with many autoimmune, autoinflammatory, and pigmentary disorders, such as type 1 diabetes, thyroid disorders, or rheumatoid arthritis [143]. The initial event in melanocyte destruction may be the occurrence of oxidative stress. Both endogenous and exogenous factors can trigger the production of reactive oxygen species (ROS). The main exogenous stress factor is UV radiation, while among the endogenous factors, the process of melanin synthesis can be distinguished due to the fact that it generates a pro-oxidant and absorbs a large amount of energy. In addition, high protein production during melanogenesis greatly increases the risk of protein misfolding, which is associated with vitiligo predisposition [142]. Melanocytes respond to stress factors by producing ROS, causing an imbalance between pro-oxidants and both enzymatic and non-enzymatic antioxidants in the blood and skin. Moreover, vitiligo melanocytes have dysregulated autophagy and are more sensitive to ROS due to the impairment of the nuclear E-2-related factor 2 pathway, which is responsible for protecting cells from oxidative stress [131,143]. Both cell-mediated and immune abnormalities have been implicated in the pathogenesis of vitiligo. Antibodies to melanocyte antigens, which can cause melanocyte destruction, have been identified in vitiligo patients. Cytotoxic CD8+ T cells found in the blood of vitiligo patients are also responsible for melanocyte destruction by inducing their apoptosis. The JAK-STAT pathway is activated by the binding of INF-γ to its receptor and leads to increased skin secretion of CXCL9 and CXCL10, which enhance the inflammatory process [141].

The most important information on hypomelanoses has been collected and presented in Table 3.

## 10. Treatment of Skin Coloration Disorders

Skin pigmentation disorders are characterized by multifactorial causes, including genetic, hormonal, post-inflammatory, and drug-induced ones, which create difficulties in removing the changes and significantly prolong the therapeutic process. A detailed analysis of the types and causes of pigmentation disorders increases the likelihood of effective prevention and treatment. Despite the fact that most pigmentation disorders are cosmetic problems, they often also affect the patient’s well-being. The basic methods of treating pigmentation disorders include both natural treatment methods and the use of oral and topical medicines. Moreover, in recent years, there has been an increase in the availability of advanced laser technologies that help eliminate pigmentation disorders [144].

### 10.1. The Treatment Options for Hyperpigmentation

One of the frequently used alternatives for the treatment of hyperpigmentation disorders are natural and plant-based medicines, which are characterized by high safety of use. The basic mechanisms of lightening agent action include inhibition of melanogenesis by decreasing the conversion of L-DOPA to melanin and inhibition of tyrosinase transcription (hydroquinone) and activity (kojic acid, arbutin, cysteamine, and corticosteroids). Moreover, topical natural products have the ability to reduce the transfer of melanosmes to keratinocytes (retinoids, niacinamide), degrade melanosomes and damage to melanocytes (hydroquinone). The inhibition of the activity of alpha-melanocyte-stimulating hormone activation and tyrosinase also contributes to the whitening effect (bakuchiol, tranexamic acid). Strong antioxidant properties are used to support the anti-hyperpigmentation properties of other compounds and to obtain a better anti-hyperpigmentation effect (ascorbic acid, cysteamine, and kojic acid). The most effective method of treating hypermelanosis is a combination of several whitening preparations with different mechanisms of action, e.g., retinoic acid, hydroquine, and corticosteroide mixtures (Kligman formula) [145].

Another well-tolerated alternative to lighten, especially post-inflammatory hyperpigmentation, involves the use of chemical peels containing 30–70% glycolic acid or 14% salicylic acid, 14% resorcinol, and 14% lactic acid (Jessner’s peel), and 10–35% trichloroacetic acid, allowing for controlled destruction of external skin layers and increased keratinocyte turnover [146].

Promising new methods for whitening skin lesions include lasers emitting a wide range of electromagnetic radiation. Intense Pulsed Light (IPL) lasers emit radiation in the range of 500–1200 nm; Q-switched nanosecond lasers (QSL) (1064 nm) have the ability to penetrate deeper layers of the skin; and Q-switched picosecond lasers (QL) are commonly used for effective whitening of skin lesions. The gripping points of laser devices are skin chromophores such as melanin and hemoglobin, which are fragmented by photochemical or photothermal conditions. Currently available data suggest the effectiveness of laser therapy is over 80%, but a full assessment of the safety of their use is limited due to the lack of randomized controlled clinical trials [137].

It is worth emphasizing that the avoidance of UV radiation and, consequently, regular daily application of photoprotection 50+ are essential elements for the success of therapy [147].

### 10.2. The Treatment Options for Hypopigmentation

The currently used strategies for vitiligo therapy include mainly phototherapy and topical treatment. The first-line option for hypopigmentation treatment is corticosteroids usage, such as mometasone furoate, clobetasol dipropionate, or betamethasone dipropionate because of their antimitogenic, immunosupperessive, and anti-inflammatory effects. The basic mechanism of action of glucocorticoids is based on NF-KB inhibition, which prevents demarginating and apoptosis of neutrophils, and phospholipaze A2 inhibition, which prevents the formation of inflammatory reaction mediator synthesis.

Nowadays, calcineurin inhibitors, e.g., tacrolimus and pimecrolimus, are also recommended for vitiligo management. Oral antioxidants—vitamin C, vitamin E, and minocycline—are used to restore the skin oxidation–antioxidant system [148]. With traditional treatment, phototherapy, including narrow-band ultraviolet (NB-UVA) and psoralen ultraviolet A (PUVA), causing apoptosis of lymphocytes, becomes an interesting alternative for vitiligo treatment. This method, used both alone and with additional drugs, is highly successful in repigmentation induction [149].

## 11. Conclusions

To summarize, the presented article focuses on the important biological aspects underlying the pigmentation of the skin, eyes, and hair follicles. The first part discusses the nature and basic biological functions of skin pigment cells and the types and properties of melanin pigments. Particular attention was paid to the influence of the structure of melanin biopolymers on the possibility of forming drug–melanin complexes and, therefore, on the possible impact on the effectiveness and safety of pharmacotherapy due to the fact that pharmacokinetic parameters may change. Secondly, hereditary and acquired pigmentation disorders—hypo- and hypermelanosis affecting the appearance and well-being of society—are presented in detail. Finally, the possibilities of therapy, including substances of natural origin, chemical peels, and laser therapy for pigmentation diseases, are presented.

## Figures and Tables

**Table 1:** The characteristics of the Fitzpatrick scale.

Skin Phototype	Sunburn Susceptibility	Tanning
I	High	None
II	High	Weak
III	Moderate	Medium
IV	Low	Dark
V	Very low	Naturally dark skin
VI	Very low	Dark brown or black skin

**Table 2:** Hereditary pigmentation disorders and the treatment possibilities.

Disease	Type of Pigmentation Disorder	Molecular Basis of Disease Development	Symptoms Related to Pigmentation Disorders	Other Symptoms Associated with the Disease	Frequency of Occurrence	Type of Inheritance
Generalized lentiginosis	Hyperpigmentation	Chromosome mutation 4q21.1-q22.3	Numerous widely distributed lentigines	Lack of other systemic abnormalities	Rare	Autosomal dominant manner
LEOPARD syndrome	Hyperpigmentation	*PTPN11* gene mutation	Numerous lentigines located mainly around the joint of the lower limbs	Abnormalities in cardiac, visual, pulmonary, reproductive systems, and deafness	Rare	Autosomal dominant manner
Carney complex	Hyperpigmentation	*PRKAR1A* gene mutation	Multiple skin and mucous lentigines	Multiple endocrine and non-endocrine neoplasms; cutaneous and cardiac myxomatous tumors	Rare	Autosomal dominant mamnner
Peutz–Jeghers syndrome	Hyperpigmentation	*STK11/LKB1* gene mutation	Mucocutaneous lips hyperpigmentations	Polyps in gastrointestinal tract; increased risk for malignant tumor development	Rare	Autosomal dominant manner
Albinism	Hypopigmentation	*TYR* gene mutation	Pale skin, hair, and eyes	Increased risk of skin cancer development	1:5000–1:40,000 cases	Autosomal recessive manner
Chediak–Higashi syndrome	Hypopigmentation	*LYST* gene mutation	Partial oculocutaneous albisim	Immune dysfunction, neurodegeneration, and hemophagocytic lymphohistiocytosis	Rare	Autosomal recessive manner
Hermansky-Pudlak syndrome	Hypopigmentation	*HPS* gene mutation	Partial oculocutaneous albinism	Pulmonary fibrosis, immunodeficiency, prolonged bleeding, and neuropsychological disorders	1–9/1,000,000 cases	Autosomal recessive manner
Waardenburg syndrome type 1 and 3	Hypopigmentation	*PAX3* gene	Irregular depigmentation areas located on the limbs, abdomen and chest, white eyebrows and eyelashes, deafness, and irises heterochromia	Face and hand malformations	2–3/100,000 cases	Autosomal dominant or recessive manner
Waardenburg syndrome type 2	Hypopigmentation	*MITF* gene mutation	White spots and pale complexion and hair	Organ defects	1/40,000 cases	Autosomal dominant or recessive manner
Waardenburg syndrome type 4	Hypopigmentation	*EDN3* gene mutation	White spots	Megacolon and neural crest defects	Rare	Autosomal dominant or recessive manner
Tietz syndrome	Hypopigmentation	*MITF* gene mutation	General depigmentation and deafness	-	Rare	Autosomal recessive manner
Griscelli syndrome (GS) type 1,2,3	Hypopigmentation	Type 1: *MYO5A* gene mutation (chromosome 15q21)Type 2: *RAB27A* gene mutation (chromosome 15q21)Type 3: *RAB* gene mutation (chromosome 2q37.3)	Depending on the type of GS, the syndrome is complete or partial hypopigmentation; lack of hair color	Type 1: severe neurological disorders, numerous developmental defects, mental retardationType 2: immunosuppression, which can develop into hemophagocytic syndromeType 3: only hypopigmentation symptoms in skin and hair	Rare	Autosomal recessive manner
Piebaldism	Hypopigmentation	*KIT* gene mutation	Lack of pigment cells in the scalp, torso, and limb areas; the borders of spots are hyperpigmented	-	1:100,000 cases	Autosomal dominant manner
Hyperpigmentation treatment methods	Hereditary cutaneous hyperpigmentations are diseases that often do not require treatment. For cosmetic reasons, laser therapy is used. Chemical peels and agents with antioxidant properties are used supportively.					
Hypopigmentation treatment methods	Primary treatments for hereditary hypopigmentation diseases include skin grafting, cell transplantation, camouflage techniques, and the use of hair dye for poliosis.					

**Table 3:** Acquired pigmentation disorders and the therapy options.

Disease	Type of Pigmentation Disorder	Molecular Basis of Disease Development	Symptoms	Affected Part of the Body	Occurrence	Treatment Possibilities
Melasma	Hyperpigmentation	The detailed pathogenesis is currently not fully elucidated. Risk factors are frequent UV exposure, genetic predispositions, and female hormone stimulation	Asymmetric irregular brown spots and patch	Mainly face (forehead, cheeks, and chin); rarely arms, chest, or neck	The areas with more intensive UV exposure (Asia, Africa, and Latin America) have significantly higher incidence rates	Topical steroids, chemical peels, dermabrasion, and microdermabrasion
Riehl melanosis	Hyperpigmentation	Allergic contact dermatitis to cosmetic product ingredients such as fragrance	Reticulate gray-brown to black spots	Face, neck, and upper chest	The vast majority of cases are found in the Asian population	Topical agents including hydroquinone, corticosteroids, retinoids, vitamin C, azelaic acid, chemical peels (trichloroacetic acid, glycolic acid), intense pulsed-light therapy, and low-fluence Q-switched lasers
Post-inflammatory hyperpigmentation	Hyperpigmentation	Inflammation leads to increased level of mediators, i.e., IL-1, IL-6, LT-C4, LT-D4, PGE2, and PGD2, whichstimulate melanogenesis process	Flat, tan, brown, or black spots on the skin	The areas exposed to UV radiation	PIH affect all skin types; however, a higher incidence is found in dark-skinned individuals	Topical agents including hydroquinone, corticosteroids, azelaic acid, vitamin C, tretionoin, and glycolic acid peels
Lentigo sensilis	Hyperpigmentation	Paracrine stimulation of melanogenesis by factors that include HGF, EDN1, KGF/FGF7, and SCF/KITL	Beige or brown spots	UV-exposed areas, i.e., face, neckline, hands, and shoulder	Mainly elderly population	Cysteamine, cryotherapy, and laser treatment
Cronkhike–Canada syndrome	Hyperpigmentation	Autoimmune disease associated with inflammation and increased IgG4 levels	Skin hyperpigmentation, numerous gastrointestinal polyps, weight loss, and alopecia	Hypopigmented lesions may involve the whole body	Rare disease; 450 cases described so far	Treatment of skin lesions is not required
Idiopathic guttate hypomelanosis	Hypopigmentation	Reduction in the total number of melanocytes, structural abnormalities of melanocytes, such as reduced tyrosinase activity, reduction in the number of melanosomes, or adjacent keratinocyte defects	Numerous oval, small, discrete, and porcelain-white macules	Sun-exposed areas, i.e., forearms	Available data show that IGH is present in approximately 80% of the population by the age of 70	Topical steroids, tacrolimus, retinoids, cryotherapy, chemical peel, excimer laser, and skin grafting
Leucoderma punctata	Hypopigmentation	Rare side effect of UVA and UVB phototherapy in patients with psoriasis or Q-switched and carbon dioxide lasers	Numerous, distinct, and round or oval depigmented spots	Chest and back, occasionally on the face	Usually occurs in fair-skinned females before the age of 40	Laser therapy narrow-band ultraviolet B (NB-UVB) and the 308 nm excimer laser
Vitiligo	Hypopigmentation	The etiopathology of vitiligo has not been elucidated, but the progressive loss of melanocytes has been linked to a number of factors: metabolic abnormalities, oxidative stress, inflammation, and autoimmunity	White macules in the skin and/or hair	There are two main types of vitiligo: generalized, a common symmetrical form, and segmental, affecting only one side of the body	Vitiligo affects 0.5–2% of the global population	Topical or systemic corticosteroids as monotherapy (in localised vitiligo), or in combination with phototherapy or other topical agents (in generalised vitiligo), calcineurin inhibitors (tacrolimus, pimecrolimus), topical vitamin D3 analogues (calcipotriol), antioxidants, and phototherapy

## Data Availability

Data sharing is not applicable.
